# Thy-1 (CD90)-Induced Metastatic Cancer Cell Migration and Invasion Are β3 Integrin-Dependent and Involve a Ca^2+^/P2X7 Receptor Signaling Axis

**DOI:** 10.3389/fcell.2020.592442

**Published:** 2021-01-12

**Authors:** Marianne Brenet, Samuel Martínez, Ramón Pérez-Nuñez, Leonardo A. Pérez, Pamela Contreras, Jorge Díaz, Ana María Avalos, Pascal Schneider, Andrew F. G. Quest, Lisette Leyton

**Affiliations:** ^1^Cellular Communication Laboratory, Program of Cellular & Molecular Biology, Center for Studies of Exercise, Metabolism and Cancer (CEMC), Instituto de Ciencias Biomédicas, Facultad de Medicina, Universidad de Chile, Santiago, Chile; ^2^Advanced Center for Chronic Diseases (ACCDiS), Faculty of Chemical and Pharmaceutical Sciences & Faculty of Medicine, Universidad de Chile, Santiago, Chile; ^3^Instituto de Ciencias Biomédicas, Facultad de Ciencias de la Salud, Universidad Autónoma de Chile, Santiago, Chile; ^4^Department of Biochemistry, University of Lausanne, Epalinges, Switzerland

**Keywords:** Thy-1 (CD90), integrin, P2X7R, trans-endothelial migration, breast cancer, melanoma, inflammation, metastasis

## Abstract

Cancer cell adhesion to the vascular endothelium is an important step in tumor metastasis. Thy-1 (CD90), a cell adhesion molecule expressed in activated endothelial cells, has been implicated in melanoma metastasis by binding to integrins present in cancer cells. However, the signaling pathway(s) triggered by this Thy-1-Integrin interaction in cancer cells remains to be defined. Our previously reported data indicate that Ca^2+^-dependent hemichannel opening, as well as the P2X7 receptor, are key players in Thy-1-α_V_β_3_ Integrin-induced migration of reactive astrocytes. Thus, we investigated whether this signaling pathway is activated in MDA-MB-231 breast cancer cells and in B16F10 melanoma cells when stimulated with Thy-1. In both cancer cell types, Thy-1 induced a rapid increase in intracellular Ca^2+^, ATP release, as well as cell migration and invasion. Connexin and Pannexin inhibitors decreased cell migration, implicating a requirement for hemichannel opening in Thy-1-induced cell migration. In addition, cell migration and invasion were precluded when the P2X7 receptor was pharmacologically blocked. Moreover, the ability of breast cancer and melanoma cells to transmigrate through an activated endothelial monolayer was significantly decreased when the β_3_ Integrin was silenced in these cancer cells. Importantly, melanoma cells with silenced β_3_ Integrin were unable to metastasize to the lung in a preclinical mouse model. Thus, our results suggest that the Ca^2+^/hemichannel/ATP/P2X7 receptor-signaling axis triggered by the Thy-1-α_V_β_3_ Integrin interaction is important for cancer cell migration, invasion and transvasation. These findings open up the possibility of therapeutically targeting the Thy-1-Integrin signaling pathway to prevent metastasis.

## Introduction

Cancer progression involves vascularization of the primary tumor, detachment of cells from the tumor matrix, migration, intravasation, adhesion to the vascular endothelium of target organs, and extravasation. Intravasation leads tumor cells into the bloodstream, and extravasation allows the passage of cells through the endothelium in order to proliferate and form secondary tumors at distant sites, in a process known as metastasis (Fares et al., 2020). The word “metastasis” is derived from Greek and means “change of place.” Thus, cell motility is a key feature of metastatic cells.

Cell motility involves a series of steps that are required to permit forward movement (Ridley et al., 2003). The molecular mechanisms commanding motility of cancer cells are controlled by environmental cues and signals generated within the tumor cells. A well-established regulator of cancer cell migration is intracellular Ca^2+^, which is controlled by various known ion channels and signal transduction pathways (Yang et al., 2009). Despite the wealth of available information relating to the control of cancer cell migration, gaps in our understanding of this process at the molecular level still remain (Fares et al., 2020).

Over the last decades, genome-wide association studies (GWASs) have successfully identified cell adhesion molecules as one of the most prominent group of genes whose expression is altered in melanoma and breast cancer cells (Akbani et al., 2015; Wittkowski et al., 2018). Among these genes, integrins stand out because they are the receptors that facilitate adhesion, migration, and invasion of cancer cells.

Once cancer cells enter the bloodstream, they adhere to the endothelial cells (EC) of blood vessels in target tissues and then extravasate to form secondary tumors (Fares et al., 2020). Saalbach and coworkers reported that in primary melanoma, as well as in subcutaneous metastases, the cell adhesion molecule of the immunoglobulin superfamily Thy-1 (CD90) is detectable in EC, but absent in healthy skin and in benign melanocytic skin lesions (nevi) (Schubert et al., 2013). Another important step in tumor formation is angiogenesis, which implies increased expression of vascular endothelial growth factor (VEGF). Importantly, both VEGF and the proinflammatory cytokine tumor necrosis factor (TNF; aliases are TNFSF2, TNFA, and TNF-alpha) are inducers of Thy-1 expression. Thy-1 is additionally implicated in melanoma metastasis by binding to α_V_β_3_ Integrin present in cancer cells, both *in vitro* (Saalbach et al., 2005) and *in vivo* (Schubert et al., 2013). Thus, cell-cell interaction between Thy-1 on activated EC and α_V_β_3_ Integrin on melanoma cells is an essential step in melanoma metastasis. So far, adhesion and cell migration induced by the Thy-1-α_V_β_3_ Integrin interaction has not been studied in cancer cells other than melanoma. Of note, the signaling pathways triggered as a consequence of this interaction have not been defined in cancer cells.

Our group has previously reported on signaling pathways governing astrocyte migration induced by Thy-1 in a model of neuron-astrocyte interaction. The neuronal membrane protein Thy-1 binds to α_V_β_3_ Integrin through a specific domain that contains an RLD tripeptide. By using Surface Plasmon Resonance (SPR) technology (Hermosilla et al., 2008) and single-molecule assay optical mini tweezers (Burgos-Bravo et al., 2018), we demonstrated a direct interaction between Thy-1 and α_V_β_3_ Integrin, with an affinity in the nM range. Integrin engaged by Thy-1 triggers astrocyte motility by molecular mechanisms we have described in detail in the past years (Hermosilla et al., 2008; Kong et al., 2013; Lagos-Cabré et al., 2019; Leyton et al., 2019). Signaling cascades triggered by this interaction involve the activation of phospholipase C gamma (PLCγ), which generates diacylglycerol and inositol trisphosphate (IP_3_). IP_3_ activates its receptor (IP_3_R) in the endoplasmic reticulum, triggering the release of Ca^2+^ from this intracellular store. Ca^2+^ is then required for the opening of Connexin-43 and Pannexin-1 hemichannels and release of ATP, which stimulates the purinergic P2X7 receptor (P2X7R) to promote Ca^2+^ entry, thus further increasing intracellular Ca^2+^ levels (Henriquez et al., 2011; Alvarez et al., 2016). Therefore, considering the importance of the Thy-1 and α_V_β_3_ Integrin interaction in melanoma progression, relevant questions are: (i) Is Thy-1-Integrin engagement relevant for the migration of other cancer cells? (ii) What is the molecular mechanism involved in cancer cell migration downstream of Thy-1-Integrin interaction? To broaden the relevance of our findings, these questions were addressed not only in a melanoma model, but also in breast cancer cells known to express α_V_β_3_ Integrin on their surface.

We show here in MDA-MB-231 human breast cancer cells and B16F10 mouse melanoma cells that Thy-1 induces migration and invasion of metastatic cancer cells in a β_3_ Integrin-dependent manner, by increasing intracellular Ca^2+^, hemichannel opening, ATP release, and P2X7R activation. Additionally, we used EA.hy926 cells as an EC model and treated these cells with TNF. Our results indicate that TNF stimulation increases Thy-1 protein levels, which promotes trans-endothelial migration (TEM) of breast cancer and melanoma cells in a β_3_ Integrin-dependent manner. Finally, we demonstrate in an *in vivo* mouse model that silencing of β_3_ Integrin completely abolishes melanoma lung metastasis.

## Materials and Methods

### Antibodies and Reagents

The anti-Thy-1 antibody was from BD Pharmingen Inc. (Franklin Lakes, NJ, United States) and anti-Hsp90 antibody, from Santa Cruz Biotechnology (Santa Cruz, CA, United States). The anti-β_3_ Integrin polyclonal antibody was from Millipore (Burlington, MA, United States) and the secondary anti-mouse and anti-rabbit antibodies coupled to horseradish peroxidase were from Bio-Rad Laboratories (Hercules, CA, United States). Protease inhibitors were from Roche Diagnostics (Risch-Rotkreuz, Switzerland). The chemiluminescent substrate (EZ-ECL) and Protein A beads were from Pierce Chemical (Rockford, IL, United States). The siRNAs -a pool of three siRNAs targeting β_3_ Integrin and a scrambled sequence used as a control- were obtained from Ambion (Austin, TX, United States). All chemicals were purchased from Sigma-Aldrich (St. Louis, MO, United States), unless otherwise indicated.

### Cell Cultures

MDA-MB-231 human breast cancer cells (ATCC, #HTB-26) were cultured in DMEM/F12 medium, B16F10 metastatic murine melanoma cells (ATCC, #CRL6475) were maintained in RPMI 1640 medium, and EA.hy926 EC (ATCC, #CRL2922) were cultured in IMEM medium. For all these types of cells, the medium was supplemented with 10% fetal bovine serum (FBS) and 1% penicillin/streptomycin. Cells were cultured at 37°C in 5% CO_2_. Cell medium and antibiotics were from GIBCO Life Technologies (Grand Island, NY, United States), and FBS was from HyClone Laboratories (Logan, UT, United States).

### Transfection of Cells

MDA-MB-231 cells and B16F10 melanoma cells were grown for 24 h in complete medium at 50–70% confluency. Transfections were performed with 30 nM of the siRNA mix against β3 Integrin (Ambion), control siRNA (Ambion) or GFP as a transfection control, using the Amaxa Nucleofector system following the manufacturer’s instructions for the VPI-1006 transfection kit (Lonza, Cologne, Germany) as reported (Alvarez et al., 2016; Maldonado et al., 2017). Cell extracts were obtained 48 h post-transfection and analyzed by western blotting. Alternatively, transfected cells were used in migration, invasion, TEM, and metastasis assays, as indicated.

### Thy-1-Fc and TRAIL-R2-Fc Preparation

The recombinant proteins Thy-1-Fc and TRAIL-R2-Fc were obtained as previously described (Leyton et al., 2001; Hermosilla et al., 2008), and coupled to Protein A for cell stimulation. These proteins were incubated with Protein A at a 10:1 ratio for 1 h, at 4°C with gentle shaking, in phosphate-buffer saline (PBS), as reported (Henriquez et al., 2011). TRAIL-R2-Fc is a fusion protein of the receptor for the soluble apoptosis-inducing ligand, TRAIL-R2 (Schneider, 2000), which is used as a control for possible non-specific effects caused by the Fc portion of the Thy-1-Fc fusion protein (Alvarez et al., 2016; Lagos-Cabré et al., 2017). Prior to each experiment involving Thy-1-Fc or TRAIL-R2-Fc treatment, cells were serum-starved for at least 30 min in serum-free medium.

### Wound-Healing Assay

Cells were seeded in 24-well plates and grown for 24 h to 70–80% confluence. Sub-confluent cell monolayers were wounded with a sterile micropipette tip, washed twice with PBS and left in serum-free medium for 30 min (time 0 h). MDA-MB-231 cells were stimulated for 16 h and B16F10 cells were stimulated for 7 h with 4 μg of either Thy-1-Fc or TRAIL-R2-Fc coupled to Protein A (0.4 μg), in serum-free medium. Where indicated, cells were pre-incubated for 30 min with either Heptanol (500 μM), Probenecid (1 mM), or a combination of both inhibitors. Alternatively, cells were pre-treated for 30 min with A438079 (100 nM) or Apyrase (3 U/ml); or pre-treated for 1 h with BAPTA-AM (5 μM) or 2-aminoethoxydiphenyl borate (2-APB, 5 μM). In order to quantify wound closure, the cell-free area was measured at time zero and after 7 or 16 h by phase contrast microscopy (Kong et al., 2013; Alvarez et al., 2016). Considering that reactive astrocytes share many features with cancer cells (see section “Discussion”), time, concentration and selection of the drugs used in this and the other assays, were chosen according to previous studies performed by our laboratory using reactive astrocytes (Alvarez et al., 2016; Lagos-Cabré et al., 2017).

### Transwell Migration Assay

Assays were performed in Boyden Chambers (Transwell Costar, Saint Louis, MO, United States, 6.5 mm diameter, 8 μm pore size), according to the manufacturer’s instructions. Briefly, the lower surfaces of the inserts were coated with 2 μg/ml fibronectin. MDA-MB-231 or B16F10 cells (5 × 10^4^) were re-suspended in serum-free medium and incubated with 4 μg of either Thy-1-Fc or TRAIL-R2-Fc coupled to Protein A (0.4 μg). Cells were added to the upper chamber of each insert and serum-free medium was added to the lower chamber. After 2 h, inserts were removed, and the cells present on the top chamber were scratched-out. Cells that migrated to the lower side of the insert membranes were fixed with 4% paraformaldehyde in PBS and stained with a 0.1% crystal violet solution in methanol 20% for 2 h at 37°C. Finally, membranes were then gently washed, dried at room temperature, mounted in Mowiol, and cells were counted using an inverted microscope. At least ten fields were evaluated per experiment to determine the number of cells that transmigrated.

### Invasion Assay

Assays were performed in Matrigel chambers (Matrigel BD, 6.5 mm diameter, 8 μm pore size), according to the manufacturer’s instructions. Cells (2 × 10^5^) were prepared in serum-free medium, stimulated with 4 μg of either Thy-1-Fc or TRAIL-R2-Fc coupled to Protein A (0.4 μg), and added to the upper chamber of each insert while serum-free medium was added to the lower chamber. Alternatively, cells were pre-incubated for 30 min with Probenecid (1 mM), Heptanol (500 μM), Apyrase (3 U/ml) or A438079 (100 nM); or pre-treated for 1 h with BAPTA-AM (5 μM) or 2-APB (5 μM). After 24 h, inserts were removed, washed with PBS and cells that migrated to the lower side of the insert membranes were fixed in cold methanol (1%) for 2 min and then stained with 1% Toluidine Blue for 2 h. Finally, cells were counted using an inverted microscope.

### Measurement of Intracellular Calcium Kinetics

MDA-MB-231 cells were seeded on 25-mm coverslips (4 × 10^5^ cells) and left to adhere for 24 h. Cells were then incubated with 5 μM Fluo-4-AM (Life Technologies, Grand Island, NY, United States) in Ringer solution (155 mM NaCl, 4.5 mM KCl, 2 mM CaCl_2_, 1 mM MgCl_2_, 5.56 mM glucose, and 5 mM Hepes pH 7.4) at 37°C for 30 min, then washed and left in the same solution. In addition, cells were pre-incubated with 2-APB (5 μM for 1 h) or Xestospongin C (Xes C, 5 μM for 4 h). During acquisition, cells were stabilized for 2 min before adding Thy-1-Fc to the cultures. Cells were stimulated with 16 μg of either Thy-1-Fc or TRAIL-R2-Fc, both coupled to Protein A (1.6 μg). Images were acquired at 2s intervals with an XM10 camera (Olympus Latin America Inc., Miami, FL, United States). Fluorescence intensity was quantified in 100 cells per condition, using the ImageJ software (National Institute of Health, Bethesda, MD, United States).^1^ Results were expressed as (F-F0)/F0, where F corresponds to the fold change in fluorescence and F0, to basal fluorescence.

### Lucifer Yellow Uptake

MDA-MB-231 cells were seeded on 25-mm coverslips (4 × 10^5^ cells) and left to adhere for 24 h. Cells were then treated with the indicated inhibitors, as for calcium measurements, or with BAPTA-AM (5 μM for 1 h), Probenecid (1 mM for 30 min), or Heptanol (500 μM for 30 min), prior to stimulation with 16 μg of either Thy-1-Fc or TRAIL-R2-Fc coupled to Protein A (1.6 μg) for 10 min. After stimulating the cells, they were incubated at 37°C with 0.5 mg/ml of Lucifer yellow in Ringer solution for 10 min and then washed twice with the same solution. Lucifer yellow fluorescence intensity was quantified in 100 cells per condition in relative units, using the region of interest (ROI) plugin of the ImageJ software, as reported (Henriquez et al., 2011; Alvarez et al., 2016). The probe was excited at 458 nm, to quantify emission between 500 and 530 nm.

### Extracellular ATP Measurements

MDA-MB-231 cells (1 × 10^4^) were seeded in 48-well plates. After 24 h, cells were incubated in serum-free media containing 100 μM of the exonuclease inhibitor ARL-67156 (Santa Cruz Biotechnology, CA, United States) for 30 min, at 37°C. Cells were then stimulated with 8 μg of Thy-1-Fc coupled to Protein A (0.8 μg) for different time periods. Next, 50 μl of culture medium were recovered and incubated in the dark with 50 μl of CellTiter-Glo^®^ reaction mix (Promega, Madison, WI, United States). Luminescence intensity was determined in a Synergy2 multi-mode reader (Biotek Instruments, Inc., Winooski, VT, United States) and the values were interpolated using a calibration curve obtained with different ATP concentrations (0.01, 0.1, 1, and 10 μM) (Henriquez et al., 2011).

### Trans-Endothelial Migration Assay

EA.hy926 cells (2.5 × 10^4^) were grown to confluency (72 h) on top of an 8 μm-pore size membrane (Transwell, BD Biosciences) (Ortiz et al., 2016). Endothelial EA.hy926 cells were pre-treated with 10 ng/ml of TNF during the last 48 h of the monolayer formation. MDA-MB-231 or B16F10 cells (5 × 10^4^) were co-transfected with control siRNA or β3 Integrin siRNA, and GFP as a transfection control in both cases. Transfected cells were seeded onto the EA.hy926 cells monolayer. After 16 h of co-incubation, non-adherent cells were washed away with PBS and the remaining cells bound to the endothelial monolayer were fixed with 4% paraformaldehyde in PBS, stained with DAPI and mounted onto glass slides. Green-labeled cells that transmigrated through the EC monolayer and the Transwell membrane were imaged by epifluorescence microscopy (IX81, Olympus) and counted.

### Metastasis Assay

B16F10 cells (2 × 10^6^) suspended in 500 μl of physiological saline solution, were injected intravenously into the tail vein of C57BL/6 mice. The animals were sacrificed 21 days post-injection. Lungs were fixed in Feketes solution (Ortiz et al., 2016) and black metastatic tissue from lung was weighed. Metastasis was expressed as the percentage of black tissue mass with respect to total lung mass after fixation of the tissue. Note that for tumor formation and metastasis assays, male and female mice were used indifferently. This study was performed according to the rules and standards established by the Bioethics Committee on Animal Research at the Faculty of Medicine, Universidad de Chile (Protocol number CBA # 0897 FMUCH).

### Western Blotting

After treatments, cells were washed with cold PBS and lysed in 0.2 mM Hepes buffer (pH 7.4) containing 0.1% SDS, phosphatase inhibitor (1 mM Na_3_VO_4_) and a protease inhibitor cocktail (10 mg/ml Benzamidine, 2 mg/ml Antipain, 1 mg/ml Leupeptin, and 1 mM PMSF). The lysate was centrifuged at 4°C for 2 min at 20,000 × *g* to obtain the supernatants. Total protein extracts were quantified using the bicinchoninic acid (BCA) method (Pierce Chemical, Rockford, IL, United States). Whole cell extracts (50 μg total protein) were separated by SDS-polyacrylamide gel electrophoresis (SDS-PAGE) on 10% gels and then transferred to nitrocellulose. Membranes were blocked with 5% milk in 0.1% Tween-Tris buffered saline (TBS) and then probed with anti-Thy-1, anti-Hsp90, or anti-β3 integrin antibodies. Secondary goat anti-rabbit IgG and goat anti-mouse IgG antibodies conjugated to horseradish peroxidase were diluted in 5% milk/TBS. Antibody binding was detected with the EZ-ECL chemiluminescence kit (Thermo Scientific, Rockford, IL, United States), using the Discovery 12iC model chemiluminescence system from Ultralum (Claremont, CA, United States). The bands obtained were digitized and analyzed using the ImageJ program.

### Statistical Analysis

Results were compared using the Kruskal–Wallis test and Dunn’s post-test with GraphPad Prism 5 software (San Diego, CA, United States). The Mann–Whitney U test was employed for paired groups. The specific test utilized for the statistical analyses is indicated in the figure legends. Values averaged from three or more independent experiments were compared. *P* values <0.05 were considered statistically significant.

## Results

### Thy-1 Promotes Migration and Invasion of Metastatic Breast Cancer Cells in a β_3_ Integrin-Dependent Manner

**FIGURE 1:**
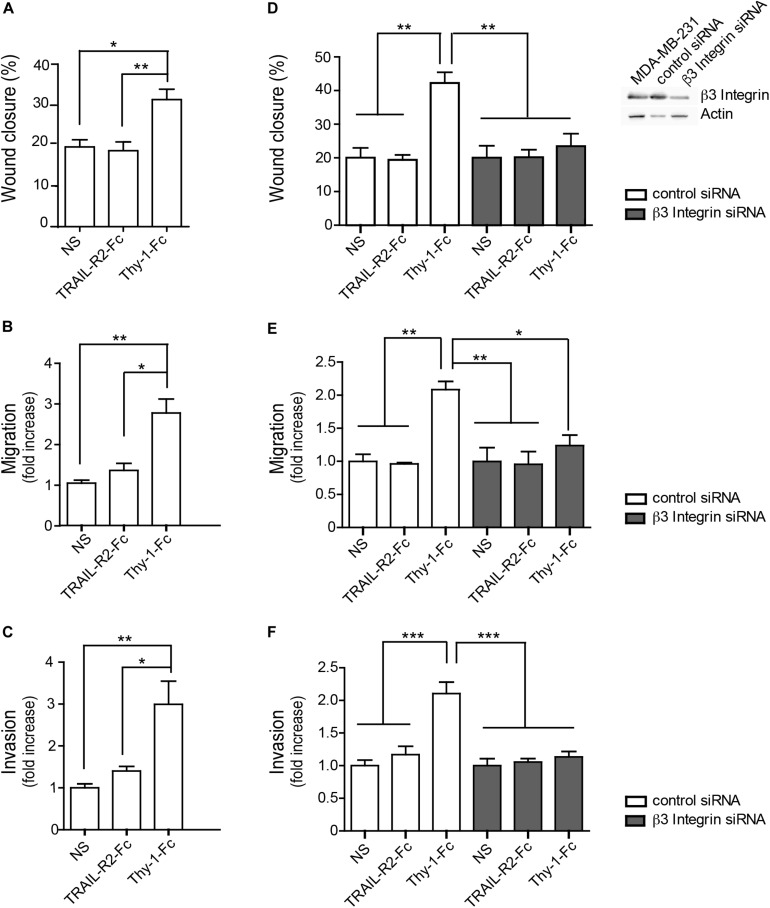
Thy-1 induces migration and invasion of MDA-MB-231 cells in a β_3_ Integrin-dependent manner. **(A)** Wound-healing assay in non-stimulated (NS), MDA-MB-231 cells, or cells treated with TRAIL-R2-Fc or Thy-1-Fc. The graph shows the quantitative analysis of cell migration after 16 h of treatment. Wound closure values (%) were obtained by estimating the cell-free area at time point 0 and after 16 h for every sample. **(B)** Transwell cell migration of MDA-MB-321 cells. Non-stimulated (NS) cells and cells stimulated with the indicated treatments were allowed to migrate for 2 h through the inserts pre-coated on the lower side with fibronectin (2 μg/ml). Migrating cells were visualized by crystal violet staining on the lower side of the inserts. Data were normalized to NS cells and are shown as the average number of cells from 30 different fields per independent experiment. **(C)** MDA-MB-231 cells were incubated as indicated in panel **(B)**, then added to Matrigel inserts, and allowed to invade for 24 h. Invasion was visualized by toluidine blue and cells were counted using an inverted microscope. Data averaged from cells counted in 20 different fields/experiment were normalized to values obtained in the NS condition. **(D–F)** Experiments were repeated as in panel **(A–C)**, respectively, using MDA-MB-231 cells transfected with control siRNA or β_3_ Integrin siRNA. Quantification of the wound-healing assay **(D)**, the Transwell migration assay **(E)**, and the cell invasion assay **(F)** of MDA-MB-231 cells, transfected and stimulated as indicated. Data shown represent the average of cells counted in 20–30 different fields from each independent experiment, normalized to the values obtained with the NS condition. Values shown in all graphs represent the mean ± SEM from three independent experiments. *p < 0.05; **p < 0.01, ***p < 0.001.

Our previous studies indicate that Thy-1 interacts with β_3_ Integrin, inducing astrocyte adhesion and migration (Leyton et al., 2001; Hermosilla et al., 2008; Avalos et al., 2009). Here, we tested whether Thy-1 promotes cell migration and invasion of metastatic breast cancer cells through its interaction with β_3_ Integrin. To this end, the MDA-MB-231 human breast cancer cell line was stimulated with Thy-1-Fc coupled to Protein A, as reported (Kong et al., 2013), and migration was evaluated in wound-healing (2D) and Boyden chamber (3D) migration assays. We found that Thy-1-Fc-stimulation of cells significantly increased wound closure and migration in the Boyden chamber assay, while for TRAIL-R2-Fc-treated [negative control (Alvarez et al., 2016; Lagos-Cabré et al., 2017)] or non-stimulated (NS) cells, no significant changes in cell migration were observed (Figures 1A,B and Supplementary Figures 1A,B). Considering that these assays were performed in serum-free medium and that the Boyden chamber assay only took 2 h, changes in cell migration are not likely due to cell proliferation. These results indicate that Thy-1 promotes the migration of MDA-MB-231 cells *in vitro.*

Enhanced cell invasion is an important process in tumor metastasis that permits colonization of secondary sites in the body (Fares et al., 2020). Thus, we assessed cell invasiveness in a Matrigel assay and found that treatment with Thy-1-Fc enhanced invasion of breast cancer cells, compared to TRAIL-R2-Fc-treated or NS controls (Figure 1C). Therefore, Thy-1 not only promotes migration, but also invasion of breast cancer cells.

We then assessed the requirement of β_3_ Integrin for breast cancer cell migration and invasion in cells in which β_3_ Integrin had been silenced. MDA-MB-231 cells were transfected with control siRNA or siRNA targeting β_3_ Integrin, and two days later they were subjected to Thy-1-Fc stimulation. Here, we demonstrate that for Thy-1-Fc-stimulated cells with silenced β_3_ Integrin protein levels (Figure 1D), wound closure and migration in the Boyden chamber assay decreased, compared with control siRNA-transfected cells (Figures 1D,E). Similar results were observed in the invasion assay; Thy-1 increased invasion of control siRNA cells, while this was not observed in cells where β_3_ Integrin had been silenced (Figure 1F). Therefore, these results show that Thy-1 promotes MDA-MB-231 cell migration and invasion in a β_3_ Integrin-dependent manner.

### Thy-1 Stimulation Activates IP_3_-Receptor Channels Increasing Intracellular Calcium Levels Required for Hemichannel Opening

**FIGURE 2:**
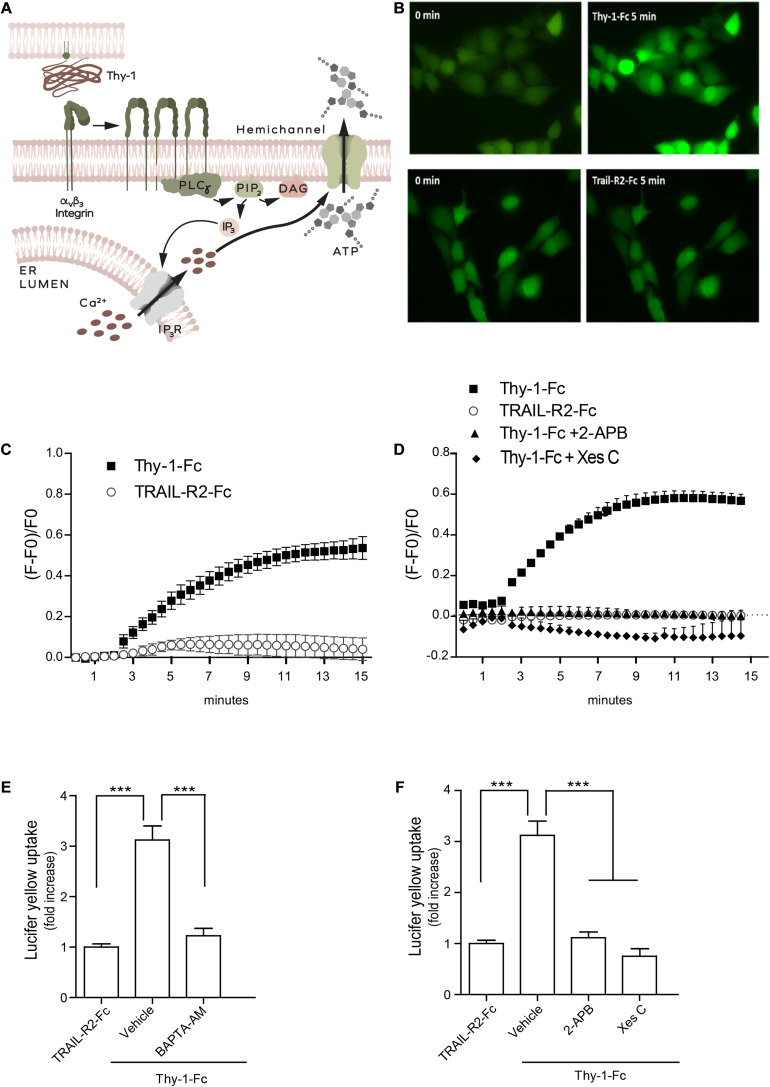
Thy-1 opens IP_3_-receptor channels, inducing intracellular Ca^2+^ increase and hemichannel opening in MDA-MB-231 cells. **(A)** Scheme of the α_V_β_3_ Integrin-triggered signaling pathway activated by Thy-1, which leads to intracellular Ca^2+^ release and hemichannel opening through the activation of PLCγ, generation of IP_3_ and activation of the IP_3_R. **(B)** Representative microphotographs of intracellular fluorescence in MDA-MB-231 cells loaded with Fluo4-AM (green) at time point = 0 and after 5 min of treatment with Thy-1-Fc or TRAIL-R2-Fc. **(C,D)** Intracellular Ca^2+^ kinetics of Fluo4-AM-loaded MDA-MB-231 cells treated with Thy1-Fc or TRAIL-R2-Fc **(C)** or stimulated with Thy1-Fc after pre-treating them with 2-APB (5 μM for 1 h) or Xestospongin C (5 μM for 4 h) **(D)**. Time point = 2 min on the graph corresponds to the moment when Thy-1 or TRAIL-R2-Fc was added to the cells in culture. Points in the graphs are mean ± SEM values obtained from three independent experiments. **(E,F)** Quantification of Lucifer yellow uptake in MDA-MB-231 cells pre-incubated with vehicle (0.001% DMSO in PBS) or BAPTA-AM (5 μM) for 1 h **(E)**, or pre-incubated with vehicle (0.001% ethanol in PBS for 1h), 2-APB, 5 μM for 1 h or Xestospongin C, 5 μM for 4 h **(F)**, and then stimulated with Thy-1-Fc. Values shown in the graph correspond to the mean fluorescence intensity (mean ± SEM) normalized to the control (TRAIL-R2-Fc condition), obtained from three independent experiments. ***p < 0.001.

Thy-1-stimulation of astrocytes triggers an intracellular signaling pathway that includes activation of PLCγ, production of IP_3_, activation of the IP_3_R, and the consequent release of calcium (Ca^2+^) from the endoplasmic reticulum (Figure 2A). If this mechanism were also relevant in cancer cells, inhibition of the IP_3_R should reduce intracellular Ca^2+^ increases and the chain of events leading to cell migration. Thus, Ca^2+^ kinetics induced by Thy-1 were analyzed with or without prior treatment with IP_3_R inhibitors. To this end, cytoplasmic Ca^2+^ elevation induced by Thy-1-Fc was measured using the Fluorescent Ca^2+^ indicator dye (Fluo-4-AM). The Ca^2+^ signal calculated from the steady-state ratio indicated that Thy-1 induced a rapid (5-7 min) and sustained intracellular Ca^2+^ release in MDA-MB-231 cells, while this was not observed in cells treated with TRAIL-R2-Fc (Figures 2B,C), for which Ca^2+^ levels remained constant. Importantly, the IP_3_R inhibitors, 2-aminoethoxydiphenyl borate (2-APB) and Xestospongin C (Xes C), completely eliminated the rise in intracellular Ca^2+^ levels stimulated by Thy-1 (Figure 2D) supporting a role for the IP_3_R in the initial release of Ca^2+^ from the endoplasmic reticulum.

Intracellular Ca^2+^ signaling has been associated with ATP release by the opening of hemichannels (Lagos-Cabré et al., 2017) and transmembrane diffusion of low molecular weight dyes has been used to indicate hemichannel opening in multiple cell types, including MDA-MB-231 cells (Qin et al., 2002). Thus, we next investigated whether Thy-1 stimulation induced the opening of hemichannels by increasing intracellular Ca^2+^ levels. We performed dye-uptake assays in MDA-MB-231 cancer cells using the low molecular weight fluorescent dye Lucifer yellow (457 Da). After stimulating the cells with Thy-1-Fc or TRAIL-R2-Fc for 5 min, we found that Thy-1-Fc induced Lucifer yellow uptake. However, this was not the case when cells were pre-incubated with an intracellular Ca^+2^ chelator, the cell permeable BAPTA-acetoxymethyl ester (BAPTA-AM) (Figure 2E). Moreover, the IP_3_R inhibitors 2-APB and Xestospongin C prevented Lucifer yellow uptake promoted by Thy-1-Fc (Figure 2F). Taken together, our results indicate that Thy-1 induces intracellular release of Ca^2+^ through IP_3_R activation, thereby increasing Ca^2+^ levels necessary for hemichannel opening.

### Thy-1-Induced Cell Migration and Invasion Require ATP Release via Hemichannel Opening and the Activation of the P2X7 Receptor

**FIGURE 3:**
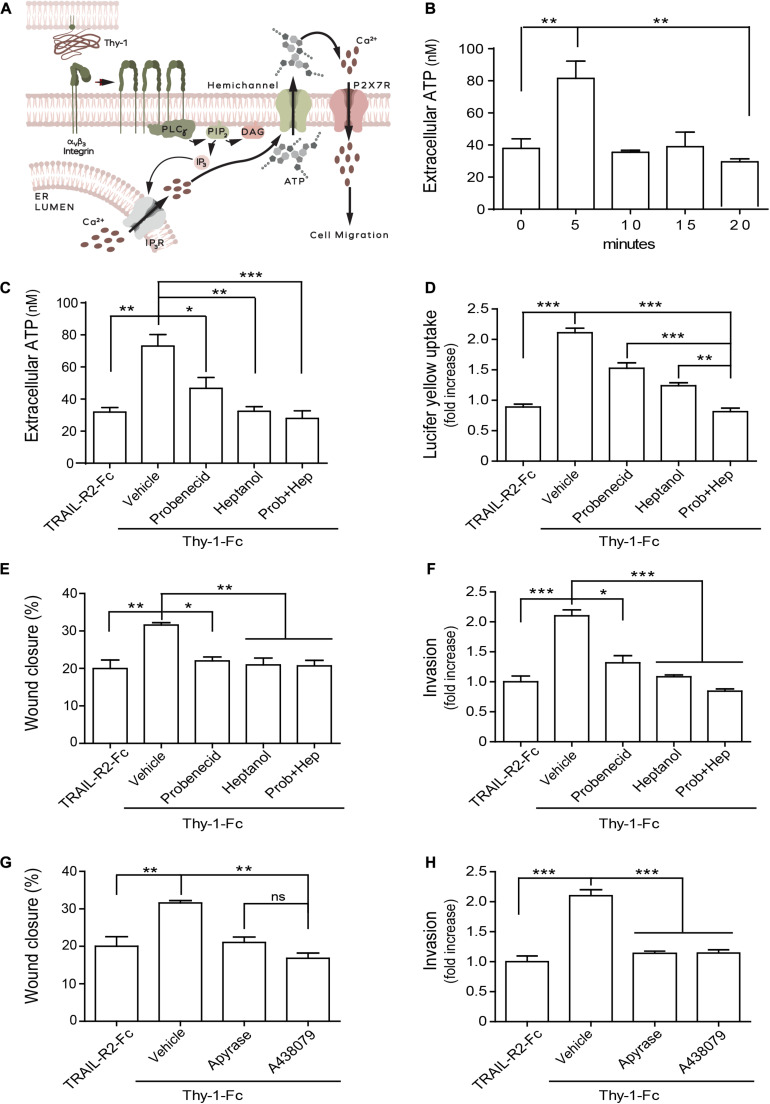
Thy-1-induced migration/invasion requires ATP release through hemichannels and the activation of the P2X7 receptor in MDA-MB-231 cells. **(A)** Schematic representation of the Thy-1-induced activation of the Ca^2+^/hemichannel opening/ATP release/P2X7R signaling pathway that initiates cell migration. **(B)** Time-dependent release of extracellular ATP in MDA-MB-231 cells induced by Thy-1-Fc during 20 min of stimulation. Quantification of: **(C)** Extracellular ATP release, **(D)** Lucifer yellow uptake, **(E)** Wound closure, and **(F)** Invasion of cells treated for 30 min with the vehicle (0.001% ethanol in PBS), the Pannexin-blocking drug Probenecid (1 mM) and/or the Connexin-blocking drug Heptanol (500 μM), and then stimulated with Thy-1-Fc for 5 min **(C)**, 15 min **(D)**, 16 h **(E)**, or 24 h **(F)**. Quantification of panel **(G)** Wound closure and **(H)** Invasion of MDA-MB-231 cells pretreated for 30 min with the vehicle (PBS), Apyrase (3 mU) or the P2X7R inhibitor A438079 (100 nM), and then stimulated with Thy-1-Fc for the times indicated in panels **(E,F)**, respectively. Data shown were normalized to values obtained with the TRAIL-R2-Fc condition used as a control. Values shown in all graphs represent the mean ± SEM from three independent experiments. ns = non-significant. *p < 0.05; **p < 0.01, ***p < 0.001.

We next evaluated whether Thy-1-stimulated MDA-MB-231 breast cancer cell migration/invasion would follow the sequence of events we have previously described for reactive astrocytes (Figure 3A; Alvarez et al., 2016; Lagos-Cabré et al., 2017), where elevated Ca^2+^ leads to hemichannel opening and ATP release. These increased levels of extracellular ATP would then promote cell migration and invasion through the activation of the P2X7R. To test this hypothesis, cells were stimulated with Thy-1-Fc for different time periods and extracellular ATP levels were measured in a colorimetric assay. We observed that treatment with Thy-1-Fc induced ATP release after 5 min of stimulation (Figure 3B). To assess whether hemichannels participated in Thy-1-induced ATP release, we used hemichannel blockers. The Pannexin inhibitor, Probenecid, the Connexin blocker, Heptanol, or a combination of both, significantly decreased extracellular ATP levels induced by Thy-1-Fc stimulation (Figure 3C). In addition, pre-treatment with Probenecid, Heptanol or a mixture of both, inhibited Lucifer yellow uptake in MDA-MB-231 cells stimulated with Thy-1-Fc (Figure 3D). The combination of both inhibitors resulted in a more significant effect than that of the individual compounds alone. Importantly, wound closure and invasion of breast cancer cells were also precluded in cells pre-treated with these hemichannel blockers (Figures 3E,F). Next, we tested whether P2X7R activation was necessary for Thy-1-induced breast cancer cell migration and invasion. Here, MDA-MB-231 cells were pretreated with the nucleotidase enzyme, Apyrase, or the P2X7R antagonist (A438079), and then stimulated with Thy-1-Fc. We found that the incubation with Apyrase precluded Thy-1-Fc-induced migration and invasion, indicating a requirement for ATP (Figures 3G,H). The same effect was observed after incubating cells with A438079 (Figures 3G,H). Altogether, these findings indicate that ATP released through hemichannels leads to the activation of the P2X7R, and that these signaling events are essential for breast cancer cell migration and invasion induced by Thy-1.

### Trans-Endothelial Migration of Cancer Cells Depends on Thy-1 Upregulation and Its Interaction With β_3_ Integrin

Trans-endothelial migration is a process that is required for tumor cells to extravasate from the vascular system and invade a specific tissue. Here, we investigated the function of β_3_ Integrin expression in TEM of cancer cells. TEM experiments were assayed on EA.hy926 cells, an immortalized hybrid of HUVEC and the A549 human lung carcinoma line. EA.hy926 is one of the most commonly used and best characterized EC lines, which exhibits many endothelium-specific properties and forms capillary-like structures in matrigel (Bauer et al., 1992). We first established a monolayer of EA.hy926 cells and then tested for monolayer permeability at different time periods (24, 48, and 72 h), as previously reported by our group (Ortiz et al., 2016). Indeed, after 72 h in culture, the dye was no longer able to permeate the cell monolayer.

**FIGURE 4:**
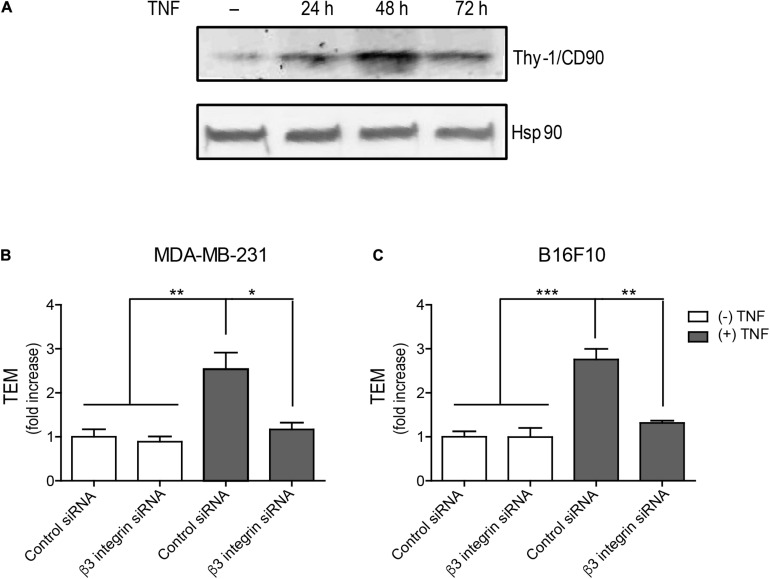
Trans-endothelial migration of MDA-MB-231 and B16F10 cells depends on Thy-1 interaction with β_3_ Integrin. **(A)** Thy-1 expression in EA.hy926 cells not treated with TNF (−) or treated with TNF during 24, 48, and 72 h. Heat Shock Protein 90 (Hsp 90) was used as a loading control. Trans-endothelial migration (TEM) of panel **(B)** MDA-MB-231 and **(C)** B16F10 cancer cells co-transfected with control siRNA (scrambled) or β_3_ Integrin siRNA and GFP, left untreated or treated with TNF (10 ng/ml, 48h). In both experiments, EA.hy926 cells were seeded on the Transwell inserts and an impermeable cell monolayer was allowed to form for 72 h. Transfected cancer cell lines (Control siRNA or β3 integrin siRNA) were added to the EA.hy926 monolayer in each insert. Cancer cells were then allowed to penetrate the EA.hy926 monolayer for 16 h. Cells that transmigrated through the endothelial monolayer were counted on the lower side of the Transwell membrane. Values in the graph correspond to the average TEM normalized to the control siRNA condition. Values shown in both graphs are the means ± SEM from three independent experiments. *p < 0.05; **p < 0.01, ***p < 0.001.

We then evaluated Thy-1 expression in EA.hy926 cells treated or not with TNF (10 ng/ml) and found that it was enhanced by TNF treatment at 24, 48, and 72 h, indicative of EC activation at these time periods, reaching a maximum after 48 h of treatment (Figure 4A). To evaluate the effect of β_3_ Integrin on MDA-MB-231 adhesion to the endothelial monolayer, the breast cancer cells were co-transfected with control siRNA or β_3_ Integrin siRNA, and GFP as a transfection control in both cases. Transfected cells were seeded onto a monolayer of EA.hy926 cells, pretreated or not with TNF (48 h), and allowed to transmigrate across the monolayer for 16 h. The ability of MDA-MB-231 cells to transmigrate through an EA.hy926 monolayer substantially increased when EA.hy926 cells were activated with TNF and was significantly reduced when the cells were transfected with β_3_ Integrin siRNA (Figure 4B). Importantly, TNF treatment for 48 h during the formation of the monolayer of EA.hy926 cells did not affect monolayer permeability (Supplementary Figure 2).

Because Thy-1 has also been implicated in melanoma metastasis by binding to integrins present in cancer cells (Schubert et al., 2013), we performed the TEM assay in B16F10 melanoma cells. Similar results were obtained when these metastatic B16F10 cells were employed; that is, TNF activation of the EA.hy926 monolayer favored TEM of B16F10 cells, but not when their β_3_ Integrin levels were reduced with siRNA (Figure 4C). Taken together, these results indicate that high levels of Thy-1 are required to promote TEM in a β_3_ Integrin-dependent manner.

### Thy-1-Induced Melanoma Cell Migration and Invasion Occur Through a β_3_ Integrin/Ca^2+^/Hemichannel/ATP/P2X7R Signaling Pathway

**FIGURE 5:**
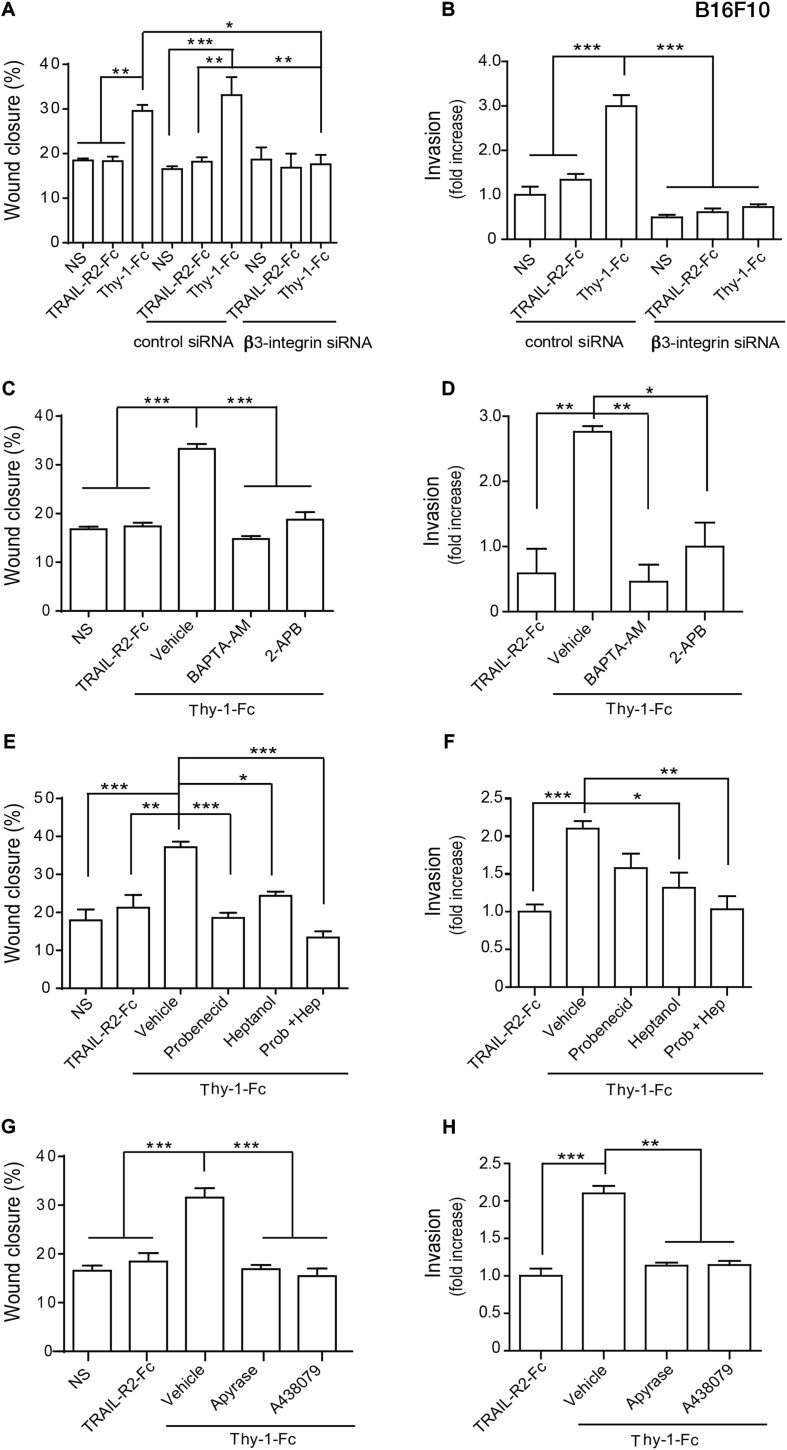
Thy-1 induces B16F10 melanoma cell migration and invasion through the β_3_ Integrin/Ca^2+^/hemichannel/ATP/P2X7R signaling pathway. **(A,B)** Non-transfected B16F10 cells and cells transfected with control siRNA or β_3_ Integrin siRNA were non-stimulated (NS), treated with TRAIL-R2-Fc or stimulated with Thy-1-Fc, and evaluated in wound closure **(A)** and invasion **(B)** assays. **(C–H)** B16F10 cells were non-stimulated (NS), treated with TRAIL-R2-Fc or pretreated with the vehicle (0.001% DMSO in PBS, 1 h), BAPTA-AM (5 μM, 1 h) or 2-APB (5 μM, 1 h) **(C,D)**; or pretreated for 1 h with the vehicle (0.001% ethanol in PBS), the Pannexin-blocking drug Probenecid (1 mM) or the Connexin-blocking drug Heptanol (500 μM), or a mixture of both drugs **(E,F)**; or pretreated for 30 min with the vehicle (PBS), Apyrase (3 mU) or the P2X7R inhibitor A438079 (100 nM), and then stimulated with Thy-1-Fc for 7 h, in order to measure wound closure **(C,E,G)** or 24 h, to assess invasion **(D,F,H)**. Values in the graph correspond to the area (as% of wound closure) at 7 h compared to Time = 0; and to the number of cells (invasion) normalized to the NS, or TRAIL-R2-Fc-treated conditions. Values shown in all graphs represent the mean ± SEM from three independent experiments. *p < 0.05; **p < 0.01; ***p < 0.001.

We then tested the involvement of β_3_ Integrin in Thy-1-induced melanoma cell migration and invasion by transfecting B16F10 melanoma cells with control siRNA or β_3_ Integrin siRNA, and then stimulating them with Thy-1-Fc. First, we corroborated that Thy-1-Fc, but not TRAIL-R2-Fc, induced migration and invasion of non-transfected B16F10 cells or cells transfected with control siRNA (Figures 5A,B). We also transfected B16F10 cells with β_3_ Integrin siRNA and observed that the Thy-1-Fc effect on their migration/invasion was dependent on the presence of β_3_ Integrin (Figures 5A,B).

The Thy-1-β_3_ Integrin interaction induces cell migration through a Ca^2+^/P2X7R signaling axis in astrocytes (Alvarez et al., 2016; Lagos-Cabré et al., 2017) and in MDA-MB-231 breast cancer cells (the present study), but whether melanoma metastasis was also dependent on Thy-1-β_3_ Integrin binding was unknown. Thus, we evaluated the participation of this signaling pathway in Thy-1-induced melanoma migration and invasion. First, the role of intracellular Ca^2+^ increase in melanoma cell migration and invasion was tested by treating B16F10 cells with BAPTA-AM or 2-APB, and then stimulating them with Thy-1-Fc. We found that these treatments precluded Thy-1-Fc-induced melanoma cell migration and invasion (Figures 5C,D). The results indicate that intracellular Ca^2+^ release is a necessary event for melanoma cell migration/invasion mediated by Thy-1, and that melanoma cell migration requires IP_3_R activation.

We also assessed hemichannel opening, ATP release and P2X7R activation in B16F10 melanoma cell migration and invasion, using the same approach as described above for MDA-MB-231 breast cancer cells. Indeed, pre-treatment of B16F10 cells with Probenecid and/or Heptanol (Figures 5E,F), Apyrase or A438079 (Figures 5G,H), all inhibited the migration/invasion of B16F10 cells, indicating the participation of hemichannels, extracellular ATP and P2X7R in melanoma cell migration/invasion.

Altogether, these results indicate that β_3_ Integrin is necessary for Thy-1-induced melanoma cell migration and invasion and that the molecular mechanisms triggered downstream of the Thy-1-β_3_ Integrin interaction include the Ca^2+^/P2X7R signaling axis that we previously described in astrocytes (Alvarez et al., 2016; Lagos-Cabré et al., 2017) and now show here for breast cancer cells.

### Silencing of β_3_ Integrin Suppresses Melanoma Cell Metastasis *in vivo*

**FIGURE 6:**
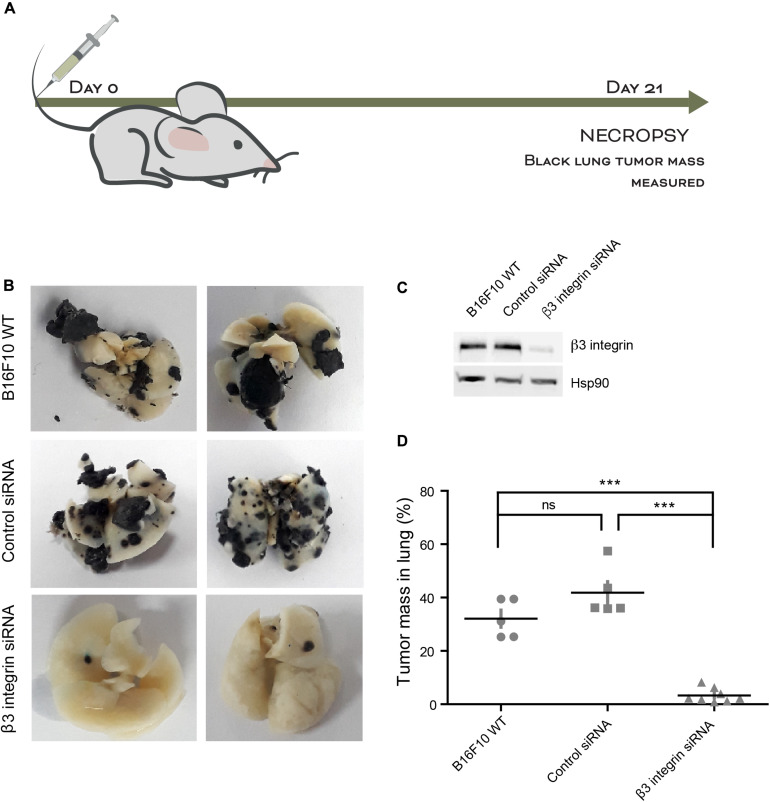
Silencing of β_3_ Integrin suppresses melanoma cell metastasis *in vivo*. **(A)** C57BL/6 mice were injected into the tail vein with non-transfected B16F10 cells (WT) or cells transfected with control siRNA or β_3_ Integrin siRNA. **(B)** Representative images of mouse lungs at 21 days post injection of WT or transfected B16F10 cells. **(C)** Quantification of tumor mass in lungs from mice treated with the indicated B16F10 cells. Values in the graph correspond to the percentage of tumor mass respect to total lung mass. ns = non-significant, ***p < 0.001.

Using a syngenic *in vivo* mouse model, we next determined whether the silencing of β_3_ Integrin expression in B16F10 cells sufficed to suppress metastasis. Non-transfected B16F10 cells or cells transfected with control siRNA or β_3_ Integrin siRNA were injected into the tail vein of C57BL/6 mice (Figure 6A). Mice were sacrificed 21 days post injection and the lung tumor mass was visualized and weighed. Dark black melanomas were observed in the lungs of mice injected with non-transfected or control siRNA-transfected cells, while almost no black nodules were detectable in the lungs of mice injected with β_3_ Integrin siRNA-transfected cells (Figure 6B), where β_3_ Integrin was knocked-down (Figure 6C). We then quantified the lung tumor mass and found that there was no significant difference between mice injected with wild type B16F10 cells and mice injected with control siRNA-transfected cells; in both cases, tumor mass was equivalent to 40% of the lung mass (Figure 6D). However, for mice injected with cells transfected with β_3_ Integrin siRNA, almost no tumors were detectable (Figure 6D), clearly indicating that β_3_ Integrin plays a key role in metastasis *in vivo*.

## Discussion

Increasing evidence points toward the involvement of Thy-1 in the regulation of cancer cell progression and metastasis (Sauzay et al., 2019). Furthermore, elevated expression of Thy-1 on blood vessel and lymphatic EC has revealed that Thy-1 participates in hematogenic and lymphogenic metastasis of melanoma cells *in vivo* (Schubert et al., 2013). Here, we show that Thy-1 induces migration and invasion of metastatic MDA-MB-231 and B16F10 cancer cells, and when upregulated in an activated EA.hy926 EC monolayer, promotes TEM of these breast cancer and melanoma cells in a β_3_ Integrin-dependent manner. We also show that β_3_ Integrin-triggered downstream signaling includes the release of Ca^2+^, hemichannel opening, ATP release, and P2X7R activation. Most importantly, silencing of β_3_ Integrin in melanoma cells (this paper) or injection of wild type B16F10 cells in Thy-1 knock-out mice (Schubert et al., 2013) abolish melanoma lung metastasis *in vivo*, indicating that disruption of the Thy-1-α_V_β_3_ Integrin interaction precludes melanoma metastases. Moreover, results obtained in the TEM assays, as well as in *in vivo* experiments, where cells were transiently transfected with β_3_ Integrin siRNA and injected two days after transfection, suggest that the Thy-1-α_V_β_3_ Integrin binding is important in an early step of the metastatic process.

Thy-1 expression is abnormally elevated in melanoma EC (Schubert et al., 2013). Intriguingly, Thy-1 is also found in malignant tumors, such as liver, esophageal, gastric, gallbladder, and lung cancer (reviewed in Kumar et al., 2016), where Thy-1 is likely to act as a tumor promoter. In contrast, Thy-1 expression is reduced in other types of cancer, such as ovarian (Abeysinghe et al., 2003), and nasopharyngeal cancer (Hong et al., 2005), suggesting that in some tumor cells, Thy-1 may also act as a tumor suppressor. Thus, the role of Thy-1 in cancer seems ambiguous, whereby tumor promoting or suppressing activities appear to depend on the cancer cell type and tumor microenvironment. Of note, our reported data in astrocyte-neuron communication have shown that the interaction between Thy-1 and α_V_β_3_ Integrin occurs both *in cis* and *in trans* (Leyton et al., 2019). The present study focuses on the effect of Thy-1 binding *in trans* with the α_V_β_3_ Integrin. However, considering that Thy-1 is reportedly present in MDA-MB-231 breast cancer cells (Wang et al., 2015), the Thy-1-integrin interaction *in cis* could also occur in these cells. In breast cancer cells, Thy-1 interaction *in cis* is likely to play a regulatory role, maintaining integrins inactive; whereas *in trans*, Thy-1 triggers a signaling cascade downstream of the integrin in the cancer cell, indicating that it leads to integrin activation. This issue of *cis* versus *trans* Thy-1-integrin interaction, and their effects on cells has been recently reviewed in Leyton et al. (2019).

Reactive astrocytes share many features with glioma cells. They proliferate, migrate, exhibit anchorage-independent growth and form neurospheres (Lang et al., 2004), express growth factors (such as VEGF), upregulate IL-6 signaling, and express elevated levels of Vimentin, Nestin, and Sox2. Levels of proteins involved in migration, proliferation, and dedifferentiation are also enhanced in reactive astrocytes (reviewed in Yang et al., 2013). In addition, and considering the importance of integrins in adhesion and migration of astrocytes (Kong et al., 2013) and cancer cells (McCabe et al., 2007; Pan et al., 2018), the similarities reported here between the mechanisms involved in migration of reactive astrocytes and tumor cells is not surprising.

Among the different integrins, β_1_ and β_3_ Integrins have essential roles in the progression of different cancer types [reviewed in Pan et al. (2018)]. Specifically, the expression of α_V_β_3_ Integrin is associated with tumor progression of human malignant melanoma (Saalbach et al., 2005) and metastatic growth of breast cancer cells in the brain (Lorger et al., 2009). Furthermore, α_V_β_3_ Integrin is an indicator of increased lymph node or bone metastasis and decreased patient survival in pancreatic, prostate, and breast cancer (Pan et al., 2018).

Different β Integrin subunits participate in the regulation of the malignant phenotype by mediating tumor cell interactions with specific extracellular matrix proteins of the tumor microenvironment. Knockdown of the β_3_ Integrin subunit was shown to reduce primary tumor growth and inhibit pancreatic cancer metastasis (Grzesiak et al., 2011). β_1_, β_3_ and β_6_ Integrin subunits are upregulated in human prostate cancer cells, which express α_V_β_3_ Integrin (reviewed in Goel et al., 2008), and the β_3_ and β_5_ subunits are differentially expressed in breast cancer cell lines (Lautenschlaeger et al., 2013). Most importantly, the analysis of colorectal cancer samples by immunohistochemistry has revealed increased levels of β_3_ Integrin expression in tumors at stage III and IV, compared with those at stages I and II (Lei et al., 2011).

By silencing β_3_ expression in melanoma cells, we demonstrate in our study that the β_3_ Integrin subunit plays a key role in *in vivo* metastasis. Besides inhibition of metastasis, loss of β_3_ Integrin may correlate with loss of tumorigenic morphology, as reported in breast cancer cells (Sesé et al., 2017). Accordingly, knockdown of endogenous β_3_ decreases anchorage-independent growth and metastasis of pancreatic cells, which may account for the enhanced malignancy associated with α_V_β_3_ Integrin expression in many tumors (Desgrosellier et al., 2009). Given the expression levels of β_3_ Integrin in many types of cancer cells, it emerges as a potentially interesting therapeutic target. However, relying only on this integrin subunit to evaluate the status of a patient would not be sufficient. Prognosis and treatment should consider, for example, the association between β_3_ and β_1_ Integrins to increase the effectiveness of targeted therapy approaches (Pan et al., 2018). Furthermore, β_3_ Integrin is critical for the communication between tumor cells and stromal cells, like fibroblasts and EC, and exhibits a cancer-promoting function via these interactions with the tumor microenvironment (Zhu et al., 2019). Therefore, future studies are required to understand the multiple roles of β_3_ Integrin in cancer, in order to progress in the design of effective targeting strategies.

On the other hand, genetic interference and pharmacological targeting of the α_*V*_ Integrin subunit might also contribute to the reduction of tumor cell function observed after silencing β_3_ Integrin. In different breast cancer lines, for example, the α_*V*_ antagonist GLPG0187 inhibits invasion and metastasis in zebrafish or a mouse xenograft model (Li et al., 2015). Due to the co-expression of α_*V*_ and β_3_ subunits in cancer cells, future studies are needed to characterize their roles in specific types of cancer. Even though integrins have been the focus for therapeutic intervention for over three decades and despite some therapeutic advances (reviewed in Janiszewska et al., 2020), their highly complex biology has often delayed drug development.

Cell migration and invasion are critical steps for the metastatic dissemination of cancer cells and metastasis (reviewed in Fares et al., 2020), which constitutes the major cause of death in cancer patients. Here, we describe how the interaction between Thy-1 (CD90) on activated ECs and β_3_ Integrin on melanoma and breast cancer cells promotes their migration, TEM, and invasion, via a signaling mechanism that involves Ca^2+^/hemichannel/ATP/P2X7R activation. Noteworthy, blocking any step of this signaling cascade inhibits cell migration, indicating the existence of a sequence of events that leads to cell motility. To move, cells create protrusions in the direction of the motion at one end, while retracting similar projections at the other end. It is a cyclical process coordinated by various proteins on the cell surface, which includes the integrin/hemichannels/P2X7R proteins and the intracellular Ca^2+^ increase. This cytosolic Ca^2+^ elevation is likely to occur at specific times and locations to modulate cell motility (reviewed in Lagos-Cabré et al., 2019).

Regulation of cancer cell migration by different signal transduction pathways has been previously reported. For example, the EP4 receptor, the predominant PGE2 receptor subtype in HT-29 and HCA-7 human colon cancer cell lines, activates PI3K and induces Ca^2+^ influx from the extracellular space through Orai1, resulting in ERK phosphorylation and migration of oral cancer cells (Osawa et al., 2020). The PI3K-AKT and Ras-ERK pathways regulate migration, invasion, and many other characteristics of cancer cells through multiple downstream effectors, establishing complex signaling networks that pose a huge challenge for the development of anticancer drugs [reviewed in Sever and Brugge (2015)]. Another possibility is that the P2Y2R, a GPCR that possesses an RGD domain and binds to activated α_V_β_3_ Integrin, could trigger downstream PLC enzyme activation, accounting for the Ca^2+^ released from intracellular stores, as reported in the present manuscript, and in astrocytes (reviewed in Henriquez et al., 2011). This possibility has never been tested in our model systems. Instead, P2X7R involvement has been demonstrated in this manuscript and in our previous reports using astrocytes (Henriquez et al., 2011; Alvarez et al., 2016). Thus, it is possible that the sustained Ca^2+^ signal reported in our studies is due to Ca^2+^ uptake through ATP-activated P2X7R, and that there is an undetectable first, sharp peak of Ca^2+^ released through the IP_3_R present in the endoplasmic reticulum. In addition, many studies have shown that the P2X7R and the tumor microenvironment play an important role in regulating the growth, apoptosis, migration and invasion of tumor cells (reviewed in Zhang et al., 2020). Involvement of the P2X7R has been described in tamoxifen-resistant MCF-7 breast cancer cells, where activity of this receptor seems to be restricted to cell migration and metastasis, rather than cell proliferation (Park et al., 2019). Therefore, in the cancer cells used in our study, the P2X7R might be selectively activated to induce cell migration. Importantly, P2X7R is present on the surface of most tumor cells (Yang et al., 2016), activates different signaling pathways (Deng et al., 2017), and promotes tumor angiogenesis (Gómez-Villafuertes et al., 2016). Therefore, reducing the expression level of P2X7R and the use of P2X7R agonists and antagonists may unveil the specific mechanism by which P2X7R participates in migration of cancer cells and other aspects of tumorigenesis.

Moreover, the effect of P2X7Rs on cell migration is still a matter of controversy, likely due to differences in local ATP concentrations. Supporting this idea is the fact that the BzATP agonist, a pharmacological agent used to stimulate P2X7R, has been shown to induce, as well as inhibit cell migration, depending on the concentration of the agonist used (Wei et al., 2008; Mankus et al., 2011; Zhou et al., 2014). Additionally, our previous studies show that BzATP alone is sufficient to mimic the effects of Thy-1-induced signaling downstream of α_V_β_3_ Integrin engagement (Henriquez et al., 2011; Alvarez et al., 2016). Perhaps, the continuous release of ATP helps maintain high local ATP concentrations, to activate P2X7R and the downstream signal transduction events required for the cyclic process of cell migration (Alvarez et al., 2016). A similar autocrine loop has been suggested to exist for the highly motile PC-9 human lung cancer cells, which show continuous ATP release and constitutive P2X7R activation in *in vitro* culture conditions (Takai et al., 2014). Moreover, the movement of dendritic cells has been attributed to P2X7R signaling in an autocrine loop that activates the opening of Pannexin-1, leading to additional ATP release (Sáez et al., 2017). This autocrine mechanism is very relevant for our studies, because we also reported a role for Pannexin-1 in ATP release in cancer cells (this study) and in astrocytes (Alvarez et al., 2016; Lagos-Cabré et al., 2017, 2018).

In summary, our study uncovers the molecular mechanisms by which cancer cell β_3_ integrin engagement of Thy-1 in EC triggers cancer cell migration and invasion, and in doing so, identifies various candidates that could be targeted to treat cancer patients, perhaps in a combined multiple-target therapy.

## Data Availability Statement

The original contributions presented in the study are included in the article/Supplementary Material, further inquiries can be directed to the corresponding author.

## Ethics Statement

The animal study was reviewed and approved by Bioethics Committee on Animal Research at the Faculty of Medicine, Universidad de Chile (Protocol number CBA # 0897 FMUCH).

## Author Contributions

MB and LL designed and executed the experiments, interpreted the data, and prepared the manuscript. SM, RP-N, LP, and PC helped to execute the experiments and interpret the data. MB, AA, and JD additionally prepared the data for publication and helped to writing the manuscript. PS, AA, AQ, and LL worked on interpreting the data and preparing the article. All authors contributed to the article and approved the submitted version.

## Conflict of Interest

The authors declare that the research was conducted in the absence of any commercial or financial relationships that could be construed as a potential conflict of interest.

## Footnotes

1 http://imagej.nih.gov/ij/

## Supplementary Material

Supplementary Figure 1 Thy-1-induced migration of MDA-MB-231 cells. **(A)** Wound-healing assay in non-stimulated (NS), or cells treated with TRAIL-R2-Fc (negative control) or Thy-1-Fc. Representative images of wound-healing assays in pseudocolor. Green cells: MDA-MB-231 cultures at 0 h. Red cells: MDA-MB-231 at 16 h after treatment. **(B)** Representative images of MDA-MB-231 cell migration in Boyden chamber. Non-stimulated (NS) cells and cells with TRAIL-R2-Fc or Thy-1-Fc treatments were allowed to migrate for 2 h through the inserts pre-coated on the lower side with fibronectin (2 μg/ml). Migrating cells were visualized by crystal violet staining on the lower side of the inserts. Magnification bar = 100 μm.

Supplementary Figure 2 *In vitro* endothelial cell permeability assay. The permeability of endothelial cell monolayers was evaluated by testing the ability of such monolayers to retain Blue dextran dye. EA.hy926 cells were grown to confluency (72 h) on top of an 8 μm-pore size membrane. Cells were pre-treated with 10 ng/ml of TNF during the last 48 h of the monolayer formation. The permeability of the monolayer was evaluated by adding complete medium with Blue dextran (10 mM) to the upper chamber and complete medium to the lower chamber. Then, after 30 min, the absorbance was determined at 618 nm. The absence of changes on the lower chamber was taken as being indicative of a sealed cell monolayer.
