# Supplementary figures and images for: Thy-1 (CD90)-Induced Metastatic Cancer Cell Migration and Invasion Are β3 Integrin-Dependent and Involve a Ca2+/P2X7 Receptor Signaling Axis
Source: Front Cell Dev Biol. 2021 Jan 12;8:592442. doi: 10.3389/fcell.2020.592442 (PMC7835543; doi:10.3389/fcell.2020.592442)

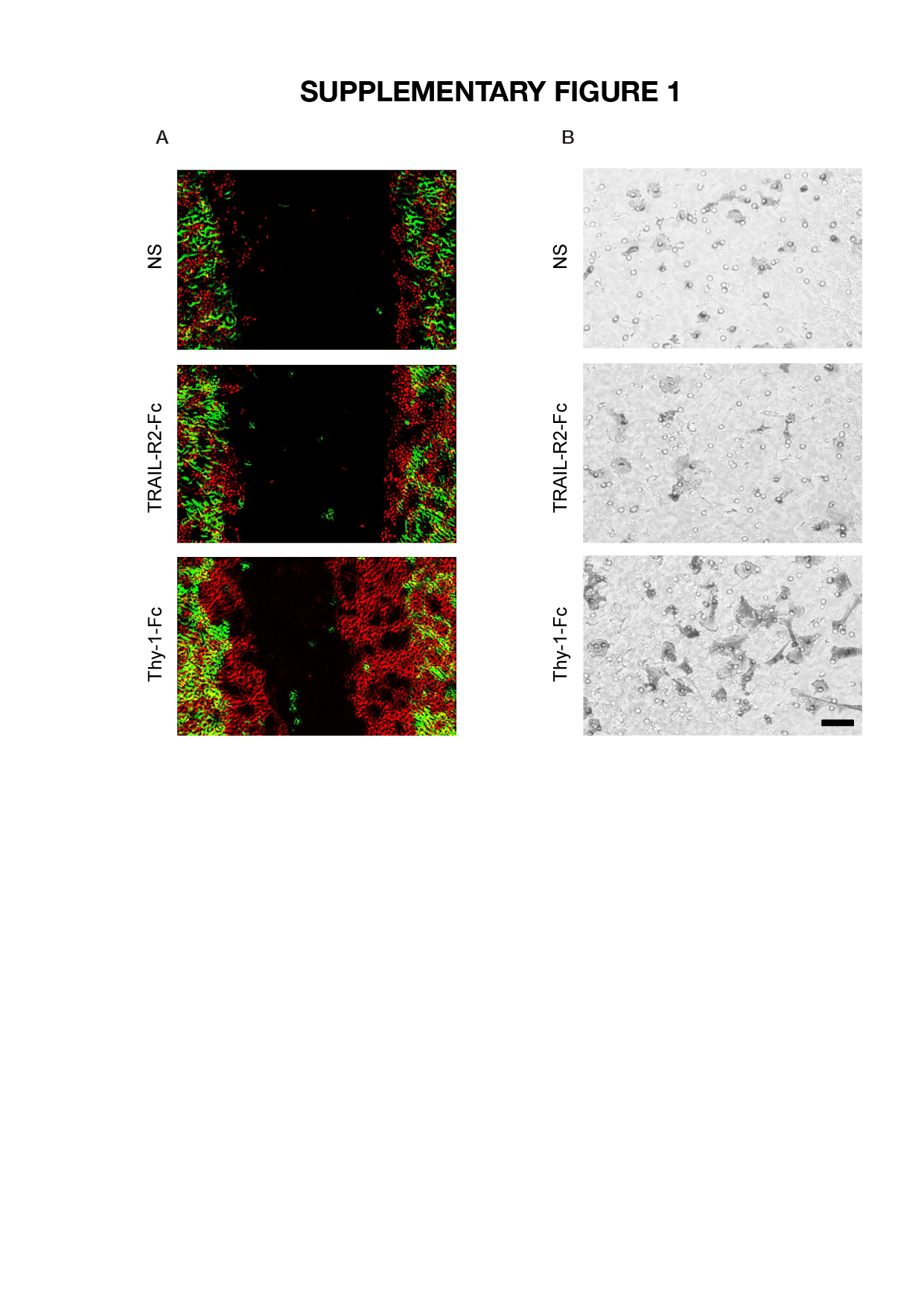

Supplement: Supplementary Figure 1 — Thy-1-induced migration of MDA-MB-231 cells. (A) Wound-healing assay in non-stimulated (NS), or cells treated with TRAIL-R2-Fc (negative control) or Thy-1-Fc. Representative images of wound-healing assays in pseudocolor. Green cells: MDA-MB-231 cultures at 0 h. Red cells: MDA-MB-231 at 16 h after treatment. (B) Representative images of MDA-MB-231 cell migration in Boyden chamber. Non-stimulated (NS) cells and cells with TRAIL-R2-Fc or Thy-1-Fc treatments were allowed to migrate for 2 h through the inserts pre-coated on the lower side with fibronectin (2 μg/ml). Migrating cells were visualized by crystal violet staining on the lower side of the inserts. Magnification bar = 100 μm. [file Image_1.TIF]

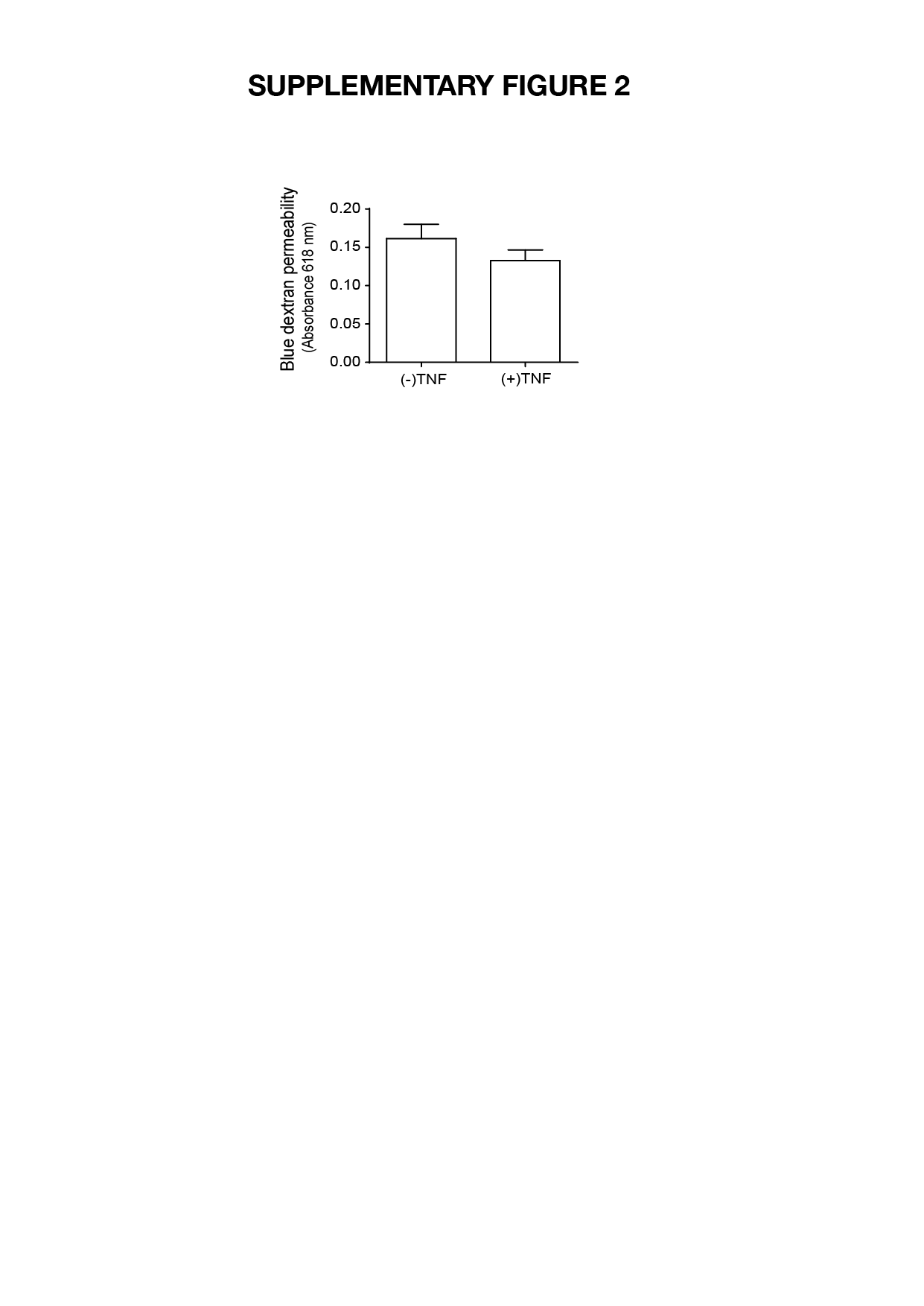

Supplement: Supplementary Figure 2 — In vitro endothelial cell permeability assay. The permeability of endothelial cell monolayers was evaluated by testing the ability of such monolayers to retain Blue dextran dye. EA.hy926 cells were grown to confluency (72 h) on top of an 8 μm-pore size membrane. Cells were pre-treated with 10 ng/ml of TNF during the last 48 h of the monolayer formation. The permeability of the monolayer was evaluated by adding complete medium with Blue dextran (10 mM) to the upper chamber and complete medium to the lower chamber. Then, after 30 min, the absorbance was determined at 618 nm. The absence of changes on the lower chamber was taken as being indicative of a sealed cell monolayer. [file Image_2.TIF]
